# Mammalian Glutamyl Aminopeptidase Genes (ENPEP) and Proteins: Comparative Studies of a Major Contributor to Arterial Hypertension

**DOI:** 10.4172/2153-0602.1000211

**Published:** 2017-06-13

**Authors:** Roger S Holmes, Kimberly D Spradling-Reeves, Laura A Cox

**Affiliations:** 1 Department of Genetics and Southwest National Primate Research Center, Texas Biomedical Research Institute, San Antonio, TX, USA; 2 Griffith Institute for Drug Design and School of Natural Sciences, Griffith University, Nathan, QLD, Australia

**Keywords:** Mammals, Glutamyl aminopeptidase, Amino acid sequence, ENPEP, Zinc metallopeptidase, Aminopeptidase A, Peptidase M1 family, Evolution, Arterial hypertensionAbbreviations: ENPEP: Glutamyl Aminopeptidase, RAS: Renin-Angiotensin System, kbps: Kilobase Pairs, CpG island: Multiple C (cytosine)-G (guanine) Dinucleotide Region, QTL: Quantitative Trait Locus, miRNA: microRNA Binding Region, BLAST: Basic Local Alignment Search Tool, BLAT: Blast-Like Alignment Tool, NCBI: National Center for Biotechnology Information, SWISS-MODEL: Automated Protein Structure Homology-modeling Server

## Abstract

Glutamyl aminopeptidase (ENPEP) is a member of the M1 family of endopeptidases which are mammalian type II integral membrane zinc-containing endopeptidases. ENPEP is involved in the catabolic pathway of the renin-angiotensin system forming angiotensin III, which participates in blood pressure regulation and blood vessel formation. Comparative ENPEP amino acid sequences and structures and ENPEP gene locations were examined using data from several mammalian genome projects. Mammalian ENPEP sequences shared 71-98% identities. Five N-glycosylation sites were conserved for all mammalian ENPEP proteins examined although 9-18 sites were observed, in each case. Sequence alignments, key amino acid residues and predicted secondary and tertiary structures were also studied, including transmembrane and cytoplasmic sequences and active site residues. Highest levels of human ENPEP expression were observed in the terminal ileum of the small intestine and in the kidney cortex. Mammalian ENPEP genes contained 20 coding exons. The human ENPEP gene promoter and first coding exon contained a CpG island (CpG27) and at least 6 transcription factor binding sites, whereas the 3′-UTR region contained 7 miRNA target sites, which may contribute to the regulation of ENPEP gene expression in tissues of the body. Phylogenetic analyses examined the relationships of mammalian ENPEP genes and proteins, including primate, other eutherian, marsupial and monotreme sources, using chicken ENPEP as a primordial sequence for comparative purposes.

## Introduction

Glutamyl aminopeptidase (ENPEP; EC 3.4.11.7; aminopeptidase A [AMPE or APA]; differentiation antigen gp160; or CD249 antigen) is one of at least 12 members of the M1 family of endopeptidases which are zinc-containing single-pass type II transmembrane enzymes [1–6]. ENPEP is involved in the catabolic pathway of the Renin-angiotensin System (RAS) forming angiotensin III, which participates in blood pressure regulation and blood vessel formation, and may contribute to risk of atrial fibrillation, angiogenesis, hypertension and tumorigenesis [7–14].

The gene encoding ENPEP (ENPEP in humans and most mammals; Enpep in rodents) is expressed at high levels in the epithelial cells of the kidney glomerulus and proximal tubule cells. ENPEP participates in the renin-angiotensin system, by way of the conversion of the biologically active Ang II (angiotensin II) to angiotensin III (Ang III), as a result of the hydrolysis of the N-terminal aspartate (or glutamate) thereby removing biological activity of the Ang peptides [15,16]. In studies of blood pressure control in hypertensive rats, ENPEP is expressed in brain nuclei where ENPEP activity generates angiotensin III, one of the major effector peptides of the brain renin angiotensin system, causing a stimulatory effect on systemic blood pressure [7,17]. Genome wide association studies have examined blood pressure variation and atrial fibrillation risk in human populations and identified an association with ENPEP variants [9,12,13,18]. In addition, studies of Enpep̄/Enpep̄ knockout mice have shown that ischemia-induced angiogenesis is impaired in these mice, as a result of decreased growth factor secretion and capillary vessel formation [8]. Other studies involved in treating hypertension in animal models using inhibitors to block ENPEP activity have also supported a direct link between ENPEP and arterial hypertension in the body [19].

Biochemical and predictive structural studies of mammalian ENPEP proteins have shown that it comprises three major domains (human ENPEP numbers quoted): An N-terminus cytoplasmic sequence (residues 1-18); a transmembrane helical sequence (residues 19-39), the signal anchor for the type II membrane protein; and an extracellular domain (residues 40-957) [1,3]. A three-dimensional protein structure has been reported for the extracellular zinc-containing endopeptidase ENPEP domain and its complexes with different ligands, which identified a calcium-binding site in the S1 pocket of ENPEP [11]. In addition, inhibitor docking studies have identified specific amino acid residues (Asp213, Asp218 and Glu215) involved in enzyme catalysis and Thr348, in performing a key role in determining substrate and inhibitor specificity for this enzyme [20].

This paper reports the predicted gene structures and amino acid sequences for several mammalian ENPEP genes and proteins, the predicted structures for mammalian ENPEP proteins, a number of potential sites for regulating human ENPEP gene expression and the structural, phylogenetic and evolutionary relationships of these mammalian ENPEP genes and proteins.

## Methods

### Mammalian ENPEP gene and protein identification

BLAST studies were undertaken using web tools from NCBI (http://www.ncbi.nlm.nih.gov/) [21,22]. Protein BLAST analyses used mammalian ENPEP amino acid sequences previously described (Table 1) [1,3,6]. Non-redundant protein and nucleotide sequence databases for several mammalian genomes were examined, including human (*Homo sapiens*), chimpanzee (*Pan troglodytes*), gorilla (*Gorilla gorilla*), orang-utan (*Pongo abelii*), colobus (*Colobus angolensis*), mangabey (*Cercocebus atys*), rhesus (*Macaca mulatta*), baboon (*Papio anubis*), snub-nosed monkey (*Rhinopithecus roxellana*), squirrel monkey (*Saimiri boliviensis*), marmoset (*Callithrix jacchus*), mouse lemur (*Microbus murinus*), cow (*Bos taurus*), sheep (*Ovis aries*), water buffalo (*Bubalus bubalis*), bison (*Bison bison*), goat (*Capra hircus*), chiru (*Pantholops hodgsonii*), camel (*Camelus ferus*), alpaca (*Vicugna pacos*), mouse (*Mus musculus*), rat (*Rattus norvegicus*), guinea pig (Cavia porcellus), horse (*Equus caballus*), pig (*Sus scrofa*), rabbit (*Oryctolagus cuniculus*), dog (*Canis familiaris*), cat (*Felis catus*), dolphin (*Tursiops truncatus*), killer whale (*Orcinus orca*) and opossum (*Monodelphis domestica*). This procedure produced multiple BLAST ‘hits’ for each of the protein and nucleotide databases which were individually examined and retained in FASTA format.

BLAT analyses were subsequently undertaken for each of the predicted ENPEP amino acid sequences using the UC Santa Cruz (UCSC) Genome Browser with the default settings to obtain the predicted locations for each of the mammalian M1 peptidase genes, including predicted exon boundary locations and gene sizes (Table 1) [23]. Structures for human isoforms (splicing variants) were obtained using the AceView website to examine predicted gene and protein structures [24]. points; the number of coding exons are listed.

### Predicted structures and properties of mammalian ENPEP M1 endopeptidases

Predicted secondary and tertiary structures for mammalian ENPEP M1 endopeptidase proteins were obtained using the SWISS-MODEL web-server (http://swissmodel.expasy.org/) [25] using the reported tertiary structure for human ENPEP [11] (PDB:4kx7A) with a modelling residue range of 76-954. Molecular weights, N-glycosylation sites, and predicted transmembrane, cytosolic and lumenal sequences for mammalian ENPEP M1 endopeptidase proteins were obtained using Expasy web tools [26,27] (http://au.expasy.org/tools/pi_tool.html). of conserved domains for ENPEP was conducted using NCBI web tools [28].

### Comparative human tissue (ENPEP) gene expression

RNA-seq gene expression across 53 selected tissues (or tissue segments) that were examined from the public database for human ENPEP, based on expression levels for 175 individuals [16] (Data Source: GTEx Analysis Release V6p (dbGaP Accession phs000424.v6.p1) (http://www.gtex.org).

### Phylogeny studies and sequence alignments

Alignments of mammalian ENPEP peptidase sequences were undertaken using Clustal Omega, a multiple sequence alignment program (Table 1) [29], Percentage identities were derived from the results of these alignments (Table 2). Phylogenetic analyses used several bioinformatic programs, coordinated using the http://www.phylogeny.fr/bioinformatic portal, to enable alignment (MUSCLE), curation (Gblocks), phylogeny (PhyML) and tree rendering (TreeDyn), to reconstruct phylogenetic relationships [30]. Sequences were identified as mammalian ENPF.P M1 endopeptidase proteins (Table 1).

## Results

### Alignments of mammalian ENPEP amino acid sequences

**Figure 1:**
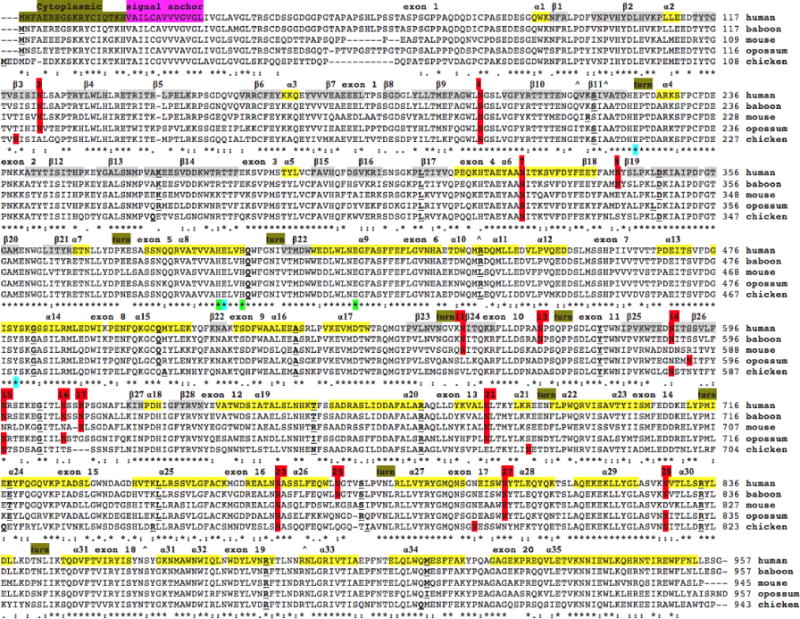
Amino acid sequence alignments for vertebrate ENPEP sequences. Table 1 for sources of ENPEP sequences; *Shows identical residues for ENPEP subunits; : Similar alternate residues;. Dissimilar alternate residues; N-glycosylated and potential N-glycosylated Asn sites are in red and numbered according to; human ENPEP active site residues are shown: Zinc binding sites, 393His, 397His, 416Glu; proton acceptor, 394Glu; and transition state stabilizer 497Tyr; other active site residues are shown as ^; α-helices for vertebrate ENPEP [11] are in shaded yellow and numbered in sequence from the N-terminus end; predicted β-sheets are in grey and similarly numbered in sequence from the N-terminus; turns in the 3D structure are shown; bold underlined font shows residues corresponding to known or predicted exon start sites; exon numbers refer to human ENPEP gene exons; four major domains were identified as cytoplasmic (N-terminal tail) (1-19); signal membrane anchor transmembrane (for linking ENPEP to the plasma membrane) (20-39; N-terminal domain (M1 aminopeptidase N) (100-545); and C-terminal domain (ERAP1-like domain) (617-931).

The deduced amino acid sequences for baboon (*Papio anubis*), mouse (*Mus musculus*), opossum (*Monodelphis domestica*) and chicken (*Gallus gallus*) ENPEP are shown in Figure 1 together with a previously reported sequence for human ENPEP [1,19] (Table 1).

**Figure 2:**
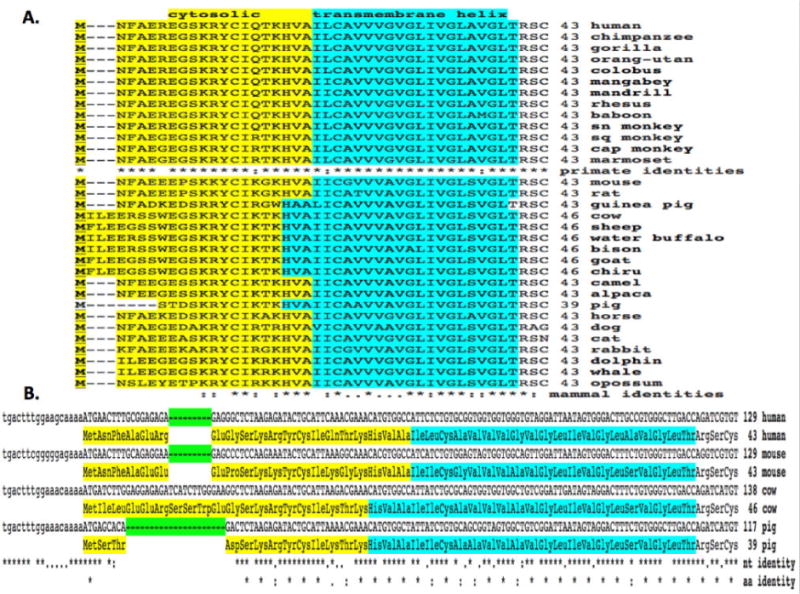
N-terminal amino acid sequence alignments (A) and 5′-nucleotide gene sequence alignments (B) for mammalian ENPEP proteins and genes. A: N-terminal mammalian ENPEP amino acid sequence alignments; *Shows identical residues for ENPEP subunits; : Similar alternate residues;. Dissimilar alternate residues; predicted cytosolic and transmembrane helical residues are shown; Table 1 for details of mammalian ENPEP proteins and genes; other mammalian ENPEP sequences were derived from NCBI as described in Methods; sn monkey: short nosed monkey; sq monkey: squirrel monkey; cap monkey: capucine monkey. B: N-Terminal mammalian ENPEP amino acid sequence alignments and 5′ mammalian ENPEP nucleotide sequence alignments; predicted cytosolic and transmembrane helical residues are shown; *Shows identical residues for ENPEP subunits and nucleotide residues; : Similar alternate residues;. Dissimilar alternate residues; ENPEP gene regions showing areas of deletions are shown.

Alignments of human and other mammalian ENPEP sequences examined were between 71-98% identical, suggesting that these are members of the same family of genes. The amino acid sequences for mammalian ENPEP proteins contained between 942 (pig) and 962 (*Mouse lemur*) amino acids, with human and most other primate ENPEP sequences containing 957 amino acids (Figures 1 and 2; Table 1).

Previous studies have reported several key regions and residues for human and mouse ENPEP proteins (human ENPEP amino acid residues were identified in each case). These included an N-terminus cytoplasmic tail (1-18) followed by a hydrophobic transmembrane 21-residue segment (19-39). A comparison of 13 primate and 19 other mammalian ENPEP sequences for these N-terminal regions revealed a high degree of conservation, particularly for residues (human ENPEP numbers used) Cys13-Ile14, His18-Val19-Ala20, Cys23, Val26, Gly30-Leu31, Val33-Gly34-Leu35 and Gly38-Leu39-Thr40-Arg41, which were invariant among all mammalian ENPEP sequences examined (Figures 1 and 2). The biochemical roles for these conserved regions include forming an N-terminal cytoplasmic tail sequence (1-19) and establishing a hydrophobic transmembrane 21-residue segment (19-39) which may anchor the enzyme to the plasma membrane [1,3,19].

Residues 41-957 of the human ENPEP sequence were identified using bioinformatics as containing two domains, including the N-terminal GluZincin Peptidase M1 (aminopeptidase N) domain (residues 100-545); and the ERAP1-like C-terminal domain (residues 617-931) [28]. The former domain includes the substrate binding site (223Glu); the Zinc binding site (1 Zinc ion per subunit) (393His, 397His, 416Glu); the proton acceptor (394Glu); and the transition state stabilizer (497Tyr) (Figure 1). The C-terminal region is predicted to be localized in the extracellular region. Five N-glycosylation sites were consistently found for all of mammalian ENPEP sequences examined, namely Asn124-Leu125-Ser126 (site 3 for mammalian sequences); Asn197-Gly198-Ser199 (site 4), Asn678-Leu679-Thr680 (site 21), Asn763-Ala764-Ser765 (site 23) and Asn801-Tyr802-Thr803 (site 27) (Figure 1 and Table 2). Other N-glycosylation sites were frequently observed for other mammalian ENPEP sequences, including Asn324-Ile325-Thr326 (site 7), Asn340-Tyr341-Ser342 (site 8), Asn554-Ile555-Thr556 (site 11), Asn567-Pro568-Ser569 (site 13), Asn589-Ile590-Thr591 (site 14), Asn597-Arg598-Ser599 (site 15), Asn607-Ser608-Ser609 (site16), Asn610-Pro611-Ser612 (site 17) and Asn828-Val829-Thr830 (site 28). One site was found among some primate ENPEP sequences, namely Asn773-Gly774-Thr775 (site 25), whereas a neighboring site (Asn796-Glu797-Thr798: site 26) was restricted to some lower primate and other mammalian ENPEP sequences (Table 2). The total number of mammalian ENPEP N-glycosylation sites differed with the species examined, from a low of 9 sites for mouse ENPEP to 18 sites for squirrel monkey and marmoset ENPEP sequences. The specific roles for ENPEP N-glycosylation sites and specific oligosaccharide residues attached to the Asparagine residues have not been determined, however given the level of conservation among different mammalian sequences examined, these are likely to play key roles in determining the physiological roles and microlocations for this enzyme in different tissues of the body.

### Predicted secondary and tertiary structures for mammalian ENPEP

**Figure 3:**
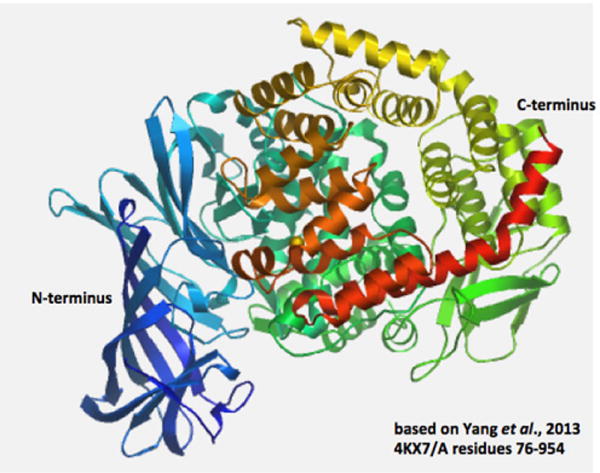
Tertiary structure for human ENPEP. The structure for human ENPEP is based on the reported structure [11] and obtained using the SWISS MODEL web site based on PDB 4KX7A (http://swissmodel.expasy.org/workspace/). The rainbow color code describes the 3-D structure from the N- (blue) to C-termini (red color); α-helices and β-sheets are shown; note the separation of 2 major domains: N-terminal M1 aminopeptidase N domain (in blue, with predominantly β-sheets); and C-terminal ERAP1-like domain (multicolored, with predominantly α-helical structures.

Predicted secondary structures for mammalian ENPEP sequences were examined, particularly for the extracellular sequences (Figure 1) using the known structure reported for human ENPEP [11] (PDB: 4kx7A), with 35 α-helices and 28 β-sheet structures being observed. Of particular interest were α-helices 8, 9 and 14 which contained the active site residues for human ENPEP. A diagram showing the tertiary structure for human ENPEP is shown in Figure 3 which demonstrates the distinct secondary structures for the N- and C-termini regions for the protein, with β-sheet structures predominating in the N-terminus region and with α-helices being the predominant structures for the C-terminus. These two major domains for human ENPEP, previously mentioned, were readily apparent, that enclose a large cavity previously shown to contain the enzyme’s active site [11]. The N-terminal domain (residues 100-545) contains the active site residues and has been recognized as a member of the peptidase M1 aminopeptidase N family, whereas the C-terminal domain (residues 617-931, recognized as an ERAP1-like domain) [31] is composed of 16 alpha helices, organized as 8 HEAT-like repeats (2 alpha helices joined by a short loop) [32], which forms a concave face facing towards the peptidase active site. This C-terminal ENPEP domain has also been shown to function as an intramolecular chaperone contributing to the correct folding, cell surface expression and activity of this enzyme [33].

### Comparative human ENPEP tissue expression

**Figure 4:**
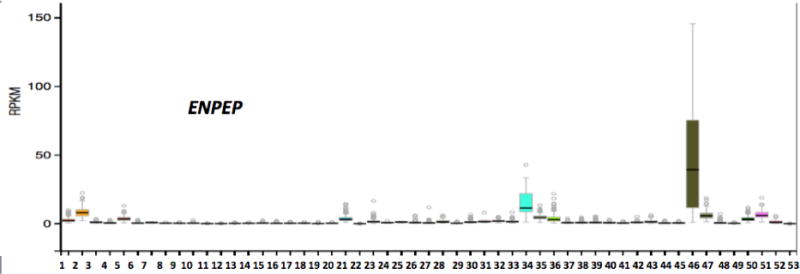
Tissue expression for human ENPEP. RNA-seq gene expression profiles across 53 selected tissues (or tissue segments) were examined from the public database for human ENPEP, based on expression levels for 175 individuals (Data Source: GTEx Analysis Release V6p (dbGaP Accession phs000424.v6.p1) (http://www.gtex.org). Tissues: 1. Adipose-Subcutaneous; 2. Adipose-Visceral (Omentum); 3. Adrenal gland; 4. Artery-Aorta; 5. Artery-Coronary; 6. Artery-Tibial; 7. Bladder; 8. Brain-Amygdala; 9. Brain-Anterior cingulate Cortex (BA24); 10. Brain-Caudate (basal ganglia); 11. Brain-Cerebellar Hemisphere; 12. Brain-Cerebellum; 13. Brain-Cortex; 14. Brain-Frontal Cortex; 15. Brain-Hippocampus; 16. Brain-Hypothalamus; 17. Brain-Nucleus accumbens (basal ganglia); 18. Brain-Putamen (basal ganglia); 19. Brain-Spinal Cord (cervical c-1); 20. Brain-Substantia nigra; 21. Breast-Mammary Tissue; 22. Cells-EBV-transformed lymphocytes; 23. Cells-Transformed fibroblasts; 24. Cervix-Ectocervix; 25. Cervix-Endocervix; 26. Colon-Sigmoid; 27. Colon-Transverse; 28. Esophagus-Gastroesophageal Junction; 29. Esophagus-Mucosa; 30. Esophagus-Muscularis; 31. Fallopian Tube; 32. Heart-Atrial Appendage; 33. Heart-Left Ventricle; 34. Kidney-Cortex; 35. Liver; 36. Lung; 37. Minor Salivary Gland; 38. Muscle-Skeletal; 39. Nerve-Tibial; 40. Ovary; 41. Pancreas; 42. Pituitary; 43. Prostate; 44. Skin-Not Sun Exposed (Suprapubic); 45. Skin-Sun Exposed (Lower leg); 46. Small Intestine-Terminal Ileum; 47. Spleen; 48. Stomach; 49. Testis; 50. Thyroid; 51. Uterus; 52. Vagina; 53. Whole Blood.

Figure 4 shows RNA-seq gene expression profiles across 53 selected tissues (or tissue segments) were examined from the public database for human ENPEP, based on expression levels for 175 individuals [16] (Data Source: GTEx Analysis Release V6p (dbGaP Accession phs000424.v6.p1) (http://www.gtex.org). These data supported highest levels of gene expression for human ENPEP in the small intestine-terminal ileum and the kidney cortex, which is consistent with the enzyme’s role in digestive tract and renal sodium (Na+) reabsorption and the renin-angiotensin system [18,34]. Lower levels were also observed in the uterus, spleen, breast, visceral adipose tissue and coronary artery, whereas brain ENPEP levels were very low according to this method, even though ENPEP has been shown to contribute to the renin angiotensin system in brain nuclei [7].

### Gene locations, exonic structures and regulatory sequences for mammalian ENPEP genes

Table 1 summarizes the predicted locations and exonic structures for mammalian ENPEP genes based upon BLAT interrogations of several mammalian and chicken genomes using the reported sequences for human and mouse ENPEP [1,8,35] and the predicted sequences for other ENPEP enzymes and the UCSC genome browser [23]. The predicted mammalian ENPEP genes were transcribed on both the negative strand (lower primates and most non-primate genomes) and the positive strand (higher primates, dog and opossum genomes). Figure 1 summarizes the predicted exonic start sites for human, baboon, mouse, opossum and chicken ENPEP genes with each having 20 coding exons, in identical or similar positions to those predicted for the human ENPEP gene. Exon 1 encodes the largest segment for each of these genes, including the cytoplasmic N-terminus and signal anchor sequences and the first 10 β-sheet structures and four of the N-glycosylation sites for mammalian ENPEP.

**Figure 5:**
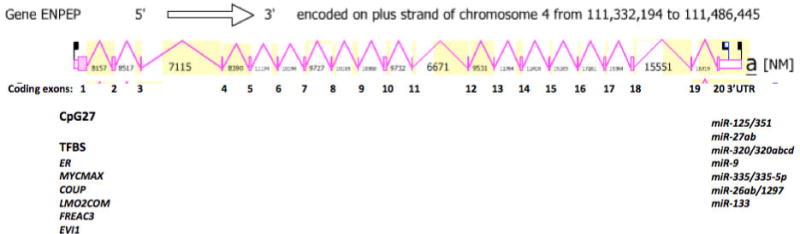
Gene structure and major gene transcript for the human ENPEP gene. Derived from the Ace View (http://www.ncbi.nlm.nih.gov/IEB/Research/Acembly/) [24]; shown with capped 5′- and 3′-ends for the predicted mRNA sequences; NM refers to the NCBI reference sequence; coding exons are in pink; the direction for transcription is shown as 5′ ? 3′; a large CpG27 island is located at the gene promoter and the first exon; predicted transcription factor binding sites (TFBS) for human ENPEP are shown; 7 predicted miRNA target sites were identified within the extended 3′-UTR region of human ENPEP.

Figure 5 shows the predicted structure for the major human ENPEP transcript together with CpG27 and several Transcription Factor Binding Sites (TFBS), which are located at the 5′ end of the gene, consistent with potential roles in regulating the transcription of this gene and forming part of the ENPEP gene promoter. The human ENPEP transcript was 4,991 bps in length with an extended 3′-untranslated region (UTR) containing 7 microRNA target sites. The human ENPEP genome sequence also contained several predicted TFBS and a large CpG island (CpG27) located in the 5′-untranslated promoter region of human ENPEP on chromosome 4. CpG27 contained 412 bps with a C plus G count of 264 bps, a C or G content of 64% and showed a ratio of observed to expect CpG of 0.64. It is likely therefore that the CpG27 Island plays a key role in regulating this gene and may contribute to the very high level of gene expression observed in the small intestine-terminal ileum and the kidney cortex [36]. At least 6 TFBS sites were colocated with CpG27 in the human ENPEP promoter region which may contribute to the high expression of this gene in human kidney and intestine.

Of special interest among these identified ENPEP TFBS were the following: The chicken ovalbumin upstream promoter transcription factor II (COUP), which has been implicated in renin gene expression, a key member of the renin-angiotensin system [37] which is highly expressed in kidney cells [38,39] the ecotropic viral integration site (EVI1) is also highly expressed in the developing kidney distal tubule and duct in *Xenopus* and plays a key role in its formation [40,41] and nuclear protein c-Myc, which plays an important role in intestinal epithelial cell proliferation [11].

It appears that the ENPEP gene promoter contains gene regulatory sequences and a large CpG island (CpG27) which may contribute to the high levels of expression observed in intestine and kidney cells. Among the microRNA binding sites observed, miR-125b has been shown to act as a tumor suppressor in breast tumorigenesis by directly targeting the ENPEP gene [10].

### Phylogeny and divergence of mammalian ENPEP M1 peptidase sequences

**Figure 6:**
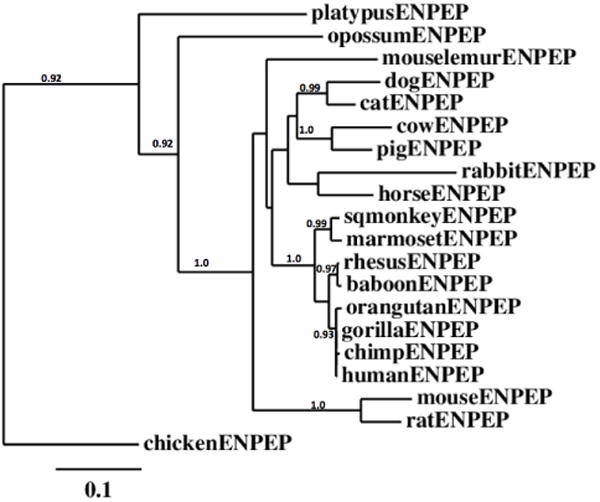
Phylogenetic tree of mammalian ENPEP amino acid sequences with the chicken ENPEP amino acid sequence. The tree is labeled with the ENPEP name and the name of the animal and is ‘rooted’ with the chicken (*Gallusi gallus*) ENPEP sequence, which was used to ‘root’ the tree (Table 1). Note the single cluster corresponding to the ENPEP gene family. A genetic distance scale is shown. The number of times a clade (sequences common to a node or branch) occurred in the bootstrap replicates are shown. Replicate values of 0.9 or more, which are highly significant, are shown with 100 bootstrap replicates performed in each case. A proposed sequence of gene evolution events is shown arising from an ancestral bird ENPEP gene.

A phylogenetic tree (Figure 6) was calculated by the progressive alignment of 19 ENPEP mammalian M1 peptidase amino acid sequences with the chicken (Gallus gallus) ENPEP sequence, which was used to ‘root’ the tree (Table 1). The phylogram showed clustering of the ENPEP sequences into groups which were consistent with their evolutionary relatedness and showing distinct groups for primate, other eutherian (mouse/rat, cow/pig and dog/cat), marsupial (opossum) and monotreme (platypus) ENPEP sequences, which were distinct from, and progressively related to each other. It is apparent that the ENPEP gene existed as a distinct mammalian gene family which has evolved from a more primitive vertebrate ENPEP gene and has been retained throughout monotreme, marsupial and eutherian mammalian evolution.

## Discussion

ENPEP is expressed at high levels in the epithelial cells of the kidney glomerulus and proximal tubule cells where the enzyme participates in the renin-angiotensin system: Renin cleaves substrate angiotensinogen forming the decapeptide angiotensin I (Ang I) [42].

1. Ang I is cleaved by Angiotensin-Converting Enzyme (ACE) to produce the biologically active angiotensin II (Ang II) [43].
2. Ang II activates its receptor (AT1) that mediates key physiological functions in the kidney (systemic regulation) and brain (central regulation), including vasoconstriction, renal sodium (Na+) reabsorption and aldosterone secretion, increasing blood pressure and contributing to hypertension [44,45].
3. Ang II is converted to angiotensin III (Ang III) by ENPEP facilitating the hydrolysis of the N-terminal aspartate (or glutamate) thereby removing biological activity of the Ang peptides [15,16].

The results of the present study indicated that mammalian ENPEP genes and encoded proteins represent a distinct gene and protein family of M1 peptidase proteins which share key conserved sequences that have been reported for other M1 peptidases previously studied [6,46,47]. Human ENPEP contains the following sites: a cytoplasmic N-terminus region (1-18); a hydrophobic transmembrane 21-residue segment (19-39), a helical signal anchor for type II membrane protein; and an extracellular protein region (residues 100-545) containing the Zinc binding endopeptidase active site (the substrate binding site (223Glu); the Zinc binding site (1 Zinc ion per subunit) (393His, 397His, 416Glu); the proton acceptor (394Glu); and the transition state stabilizer (497Tyr); and the ERAP1-like C-terminal domain (residues 617-931) (Figure 1) [28], which contain a large number of N-glycosylation sites, several of which are conserved throughout mammalian evolution. ENPEP plays a role in the catabolic pathway of the renin-angiotensin system and is a major contributor to the development of clinical arterial hypertension in the body [13,15,18,19,42,45].

## Conclusion

ENPEP is encoded by a single gene among the mammalian genomes studied and is highly expressed in human small intestine-terminal ileum and kidney cortex cells, and usually contained 20 coding exons on the negative (lower primate and other mammalian) or positive (higher primate) strands, depending on the mammalian genome. The human ENPEP gene contained a large CpG island within the promoter region, as well as several transcription factor binding sites, which may contribute to the high level of gene expression in intestinal and kidney tissues. Alignments of mammalian ENPEP sequences demonstrated the high degree of conservation observed, particularly for those regions directing the catalytic functions and structural integrity for this enzyme, especially the extracellular sequences, containing two domains, including the N-terminal GluZincin Peptidase M1 (aminopeptidase N) domain (residues 100-545); and the ERAP1-like C-terminal domain (residues 617-931). Phylogenetic studies using 19 ENPEP mammalian M1 endopeptidase sequences indicated that the ENPEP gene existed as a distinct family which has apparently evolved from a more primitive vertebrate ENPEP gene which has been retained throughout monotreme, marsupial and eutherian mammalian evolution [48–53].

## Figures and Tables

**Table 1:** Mammalian and chicken ENPEP genes and proteins.

ENPEP Gene	Species	Chromosome location	Exons#(strand)	Gene Size bps	GenBank ID*	UNIPROT ID	Amino acids	Subunit M (pI)
Human	Homo sapiens	4:110,476,415-110,561,555	20 (+ve)	85141	NM_0019977	Q07075	957	109,244 (5.3)
Chimpanzee	Pan troglodytes	4:113,095,101-113,180,147	20 (+ve)	85047	*XP_5117397	H2QQ15	957	109,115 (5.3)
Gorilla	Gorilla gorilla	4:121,992,414-122,077,571	20 (+ve)	85158	*XP_018880573	G3SK36	957	109,262 (5.3)
Orang-utan	Pongo abelii	4:115,175,027-115,261,670	20 (+ve)	86644	NM_001132893	H2PE46	957	109,098 (5.2)
Rhesus	Macaca mulatta	5:109,436,911-109,519,173	20 (+ve)	82263	NM_001266656	F7GTW9	957	109,188 (5.2)
Baboon	Papio anubis	5:101,625,577-101,709,020	20 (+ve)	83444	*XP_003899143	A0A096MTU4	957	109,192 (5.3)
Squirrel monkey	Saimiri boliviensis	*JH378138:4,950,114-5,038,89 0	20 (−ve)	88777	*XP_003929505	Na	957	109,059 (5.2)
Marmoset	Callithrix jacchus	3:83,132,279-83,220,293	20 (−ve)	88015	*XP_002806699	na	957	109,299 (5.4)
Mouse lemur	Microbus murinus	*KQ053609v1:1,352,189-1,436, 783	20 (−ve)	84595	*XP_012621645	na	962	109,104 (5.6)
Mouse	Mus musculus	3:129,270,282-129,332,481	20 (−ve)	62200	NM_007934	P16406	945	107,956 (5.3)
Rat	Rattus norvegicus	2:252,992,139-253,065,721	20 (−ve)	73583	*CH473952	P50123	945	107,995 (5.2)
Cow	Bos taurus	6:16,067,640-16,146,013	20 (−ve)	78374	NM_001038027	F1MEM5	956	109,801 (5.1)
Horse	Equus caballus	2:115,349,261-115,422,839	20 (−ve)	73579	*XP_001502921	F6XRR6	948	108,220 (4.8)
Pig	Sus scrofa	8:119,969,527-120,060,884	20 (−ve)	91358	NM_214017	Q95334	942	108,284 (5.1)
Rabbit	Oryctolagus cuniculus	15:38,927,056-39,017,176	20 (−ve)	90121	*XP_002717229	G1TBB2	956	109,013 (5.0)
Dog	Canis familiaris	32:30,553,200-30,638,483	20 (+ve)	85284	*XP_535696	F6XRM5	954	109,202 (5.4)
Cat	Catus felis	B1:113,256,430-113,341,776	20 (−ve)	85347	*XP_003985130	M3VU18	952	109,480 (5.7)
Opossum	Monodelphis domestica	5:63,362,365-63,488,028	20 (+ve)	125664	*XP_001363921	F6TL25	957	110,151 (5.4)
Platypus	Ornithorhynchus anatinus	*DS181320v1:1,408,807-1,485, 704	20 (+ve)	76898	*XP_001506613	F7E6Z3	938	107,447 (5.6)
Chicken	Gallus gallus	4:57,435,632-57,469,043	20 (−ve)	33412	*XP_426327	A0A1D5PAZ7	943	107,918 (5.0)

RefSeq: The reference amino acid sequence;

* Predicted NCBI-derived amino acid sequence; na: Not Available; GenBank IDs are derived from NCBI http://www.ncbi.nlm.nih.gov/genbank/; UNIPROT refers to UniprotKB/Swiss-Prot IDs for individual ENPEP proteins (http://kr.expasy.org); *JH and *KQ refer to a scaffold; bps refers to base pairs of nucleotide sequences; pI refers to theoretical isoelectric.

**Table 2:** Predicted locations of N-glycosylation sites for mammalian ENPEP proteins. The predicted N-glycosylation sites were numbered following alignments using Clustal Omega [29] from the N-terminal end; conserved N-glycosylation sites for all mammalian ENPEP sequences examined are highlighted in yellow; individual amino acid residues were identified using standard single letter nomenclature: N-asparagine; S-serine; T-threonine etc.

Site No	Human	Chimp	Gorilla	Orangutan	Rhesus	Baboon	Squirrel Monkey	Marmoset	Mouse Lemur	Mouse	Rat	Cow	Horse	Pig	Rabbit	Cat	Dog	Opossum
1																43NHS		44NTS
2														110NIS				
3	124NLS	124NLS	124NLS	124NLS	124NLS	124NLS	124NLS	124NLS	122NLS	116NLS	116NLS	126NVS	115NVS	114NVT	123NVS	119NVS	121NVS	123NVT
4	197NGS	197NGS	197NGS	197NGS	197NGS	197NGS	197NGS	197NGS	202NGS	189NGS	189NGS	199NGS	188NGS	187NGS	196NGS	192HGS	194NGS	197NGS
5											236NIS						241NIS	
6					272NRT		272NRT	272NRT								267NRT	269NRT	272NRT
7	324NIT	324NIT	324NIT	324NIT	324NIT	324NIT	324NIT	324NIT	329NIT	316NIT	316NIT	326NIT	315NIT	314NIT	323NIT		321NIT	324NIT
8	340NYS	340NYS	340NYS	340NYS	340NYS	340NYS	340NYS	340NYS	345NYS				331NYS					
9									383NES				367NES	367NES				
10													545NLS					
11	554NIT	554NIT	554NIT	554NIT	554NIT	554NIT	554NIT	554NIT		546NIT	547NVT	556NIT				549NIT	551NIT	
12													558NSS	557NSS		562NSS	564NLS	
13	567NPS	567NPS	567NPS	567NPS	567NPS	567NPS	567NPS	567NPS	567NPS									
14	589NIT	589NIT	589NIT	589NIT	589NIT	589NIT	589NIT	589NIT	589NIT		584NIT		580NVS	579NES	588NES	584NVS	586NVS	592NIT
15	597NRS	597NRS	597NRS	597NRS	597NRS	597NRS	597NRS	597NRS	602NRS			599NRS	588NRS	587NRS	596NRS	592NRS	594NRS	597NRT
16	607NSS	607NSS		607NSS	607NSS	607NSS	607NSS	607NSS	612NSS	601NLS	601NLS		601NPS	597NSS	606NPS	602NSS	604NSS	607NST
17	610NPS	610NPS	610NPS	610NPS	610NPS	610NPS	610NPS	610NPS	615NPS									
18												643NLS	633NLS	632NLS	641NLS	637NLS	646NFS	
19										637NHT		647NHT						646NHT
20							649NFS	649NFS	647NFS		640NFS							
21	678NLT	678NLT	678NLT	678NLT	678NLT	678NLT	678NLT	678NLT	683NLT	669NLT	669NLT	679NLT	669NLT	668NLT	677NLT	672NLT	675NLT	678NLT
22								734NDT	734NDT									
23	763NAS	763NAS	763NAS	763NAS	763NAS	763NAS	763NAS	763NAS	768NAS	754NAS	754NAS	764NAS	754NAS	753NAS	762NAS	758NAT	760NAT	763NAS
24											766NES							
25	773NGT	773NGT	773NGT		773NGT	773NGT												
26							796NET	796NET	801NET			797NET	787NET	786NET	795NET	791NET	793NET	
27	801NYT	801NYT	801NYT	801NYT	801NYT	801NYT	801NYT	801NYT	806NYT	792NYT	792NYT	802NYT	792NYT	791NYT	800NYT	796NYT	798NYT	800NYT
28	828NVT	828NVT	828NVT	828NVT	828NVT	828NVT	828NVT	828NVT				829NVT	819NVT		827NVT	823NVT	825NVT	827NVT
Total	15	15	14	14	16	15	18	18	16	9	12	12	15	14	12	15	16	13
